# 2-Oxo-1,2-dihydro­pyrimidin-3-ium di-μ-chlorido-bis­{dichloridobis[pyrimidin-2(1*H*)-one-κ*N* ^3^]cuprate(II)} dihydrate

**DOI:** 10.1107/S1600536808017455

**Published:** 2008-06-13

**Authors:** Mukhtar A. Kurawa, Christopher J. Adams, A. Guy Orpen

**Affiliations:** a School of Chemistry, University of Bristol, Bristol BS8 1TS, England

## Abstract

The asymmetric unit of the title compound, (C_4_H_5_N_2_O)_2_[Cu_2_Cl_6_(C_4_H_4_N_2_O)_2_]·2H_2_O, consists of one cation, one half of a centrosymmetric dianion and one water mol­ecule. The centrosymmetric dianion formed by dimerization in the crystal structure has neutral pyrimidin-2-one ligands coordinated to each copper(II) centre through Cu—N bonds. The Cu atoms each have a distorted trigonal bipyramidal geometry, with the N atom of the pyrimidin-2-one ligand in an axial position, and dimerize by sharing two equatorial Cl atoms. N—H⋯Cl, O—H⋯Cl and N—H⋯O hydrogen bonds connect the anions, cations and water mol­ecules, forming a three-dimensional network.

## Related literature

The anion has an essentially similar coordination environment to that of the related compound [{(C_5_H_5_N)NH_2_}CuCl_3_]_2_ which has 3-amino­pyridinium cations (Blanchette & Willett, 1988 ▶) as the nitro­gen donors and is thus neutral, while the crystal structure of the cation was described by Furberg & Aas (1975 ▶) as its chloride salt.
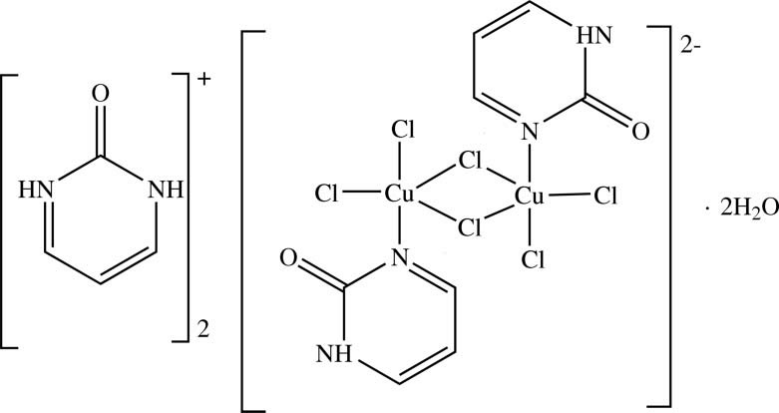


## Experimental

### 

#### Crystal data

(C_4_H_5_N_2_O)_2_[Cu_2_Cl_6_(C_4_H_4_N_2_O)_2_]·2H_2_O
*M* *_r_* = 762.22
Triclinic, 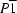

*a* = 7.5924 (4) Å
*b* = 8.6401 (3) Å
*c* = 10.6349 (4) Å
α = 96.032 (3)°
β = 100.508 (4)°
γ = 102.035 (4)°
*V* = 663.39 (5) Å^3^
*Z* = 1
Mo *K*α radiation
μ = 2.26 mm^−1^
*T* = 100 (2) K
0.41 × 0.18 × 0.15 mm

#### Data collection

Oxford Diffraction Gemini R Ultra diffractometer
Absorption correction: multi-scan (*CrysAlis RED*; Oxford Diffraction, 2007 ▶) *T*_min_ = 0.433, *T*_max_ = 0.71
14528 measured reflections
3902 independent reflections
3269 reflections with *I* > 2σ(*I*)
*R*_int_ = 0.020

#### Refinement

*R*[*F* ^2^ > 2σ(*F* ^2^)] = 0.024
*wR*(*F* ^2^) = 0.069
*S* = 1.12
3902 reflections
180 parameters
2 restraints
H atoms treated by a mixture of independent and constrained refinement
Δρ_max_ = 0.49 e Å^−3^
Δρ_min_ = −0.57 e Å^−3^

### 

Data collection: *CrysAlis CCD* (Oxford Diffraction, 2007 ▶); cell refinement: *CrysAlis RED* (Oxford Diffraction, 2007 ▶); data reduction: *CrysAlis RED*; program(s) used to solve structure: *SHELXS97* (Sheldrick, 2008 ▶); program(s) used to refine structure: *SHELXL97* (Sheldrick, 2008 ▶); molecular graphics: *SHELXTL* (Sheldrick, 2008 ▶); software used to prepare material for publication: *SHELXTL*).

## Supplementary Material

Crystal structure: contains datablocks I, global. DOI: 10.1107/S1600536808017455/sg2250sup1.cif

Structure factors: contains datablocks I. DOI: 10.1107/S1600536808017455/sg2250Isup2.hkl

Additional supplementary materials:  crystallographic information; 3D view; checkCIF report

## Figures and Tables

**Table 1:** Selected bond lengths (Å)

Cu1—N1	1.9989 (12)
Cu1—Cl3	2.2809 (4)
Cu1—Cl1	2.2830 (4)
Cu1—Cl2^i^	2.3942 (4)
Cu1—Cl2	2.6093 (4)

Symmetry code: (i) 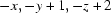.

**Table 2:** Hydrogen-bond geometry (Å, °)

*D*—H⋯*A*	*D*—H	H⋯*A*	*D*⋯*A*	*D*—H⋯*A*
N2—H2*B*⋯Cl1^ii^	0.86	2.56	3.4143 (14)	171
N3—H3*A*⋯O3^iii^	0.86	1.86	2.7099 (18)	168
N4—H2*A*⋯Cl2^iv^	0.86	2.30	3.1336 (14)	165
O3—H1⋯Cl1^v^	0.815 (16)	2.428 (17)	3.2258 (13)	166 (2)
O3—H2⋯Cl3^vi^	0.844 (17)	2.454 (18)	3.2653 (13)	162 (2)

Symmetry codes: (ii) 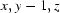; (iii) 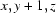; (iv) 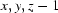; (v) 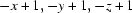; (vi) 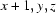.

**Fig. 1.**
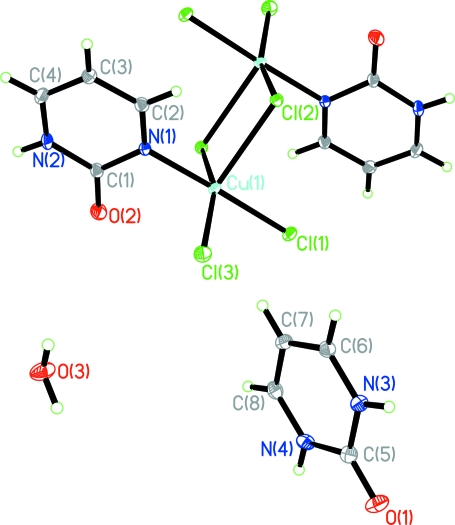
The molecular structure of I showing one dimeric dianion, one cation and a molecule of water of crystallization, with atom labels and 50% probability displacement ellipsoids for non-H atoms.

**Fig. 2.**
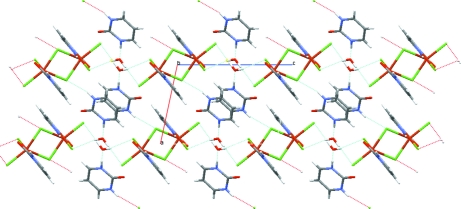
Packing of I in the ac plane, with O—H···Cl bridges between the water molecules and the dianions, N—H···O hydrogen bonds between cations and water molecules, and N—H···Cl hydrogen bonds between the anions and cations.

## supplementary crystallographic information

### Comment

N—H···Cl interactions have been extensively used in crystal engineering to design and synthesize materials with desired structures. We sought to further utilize these interactions by reacting 2-hydroxypyrimidine hydrochloride and copper(II) chloride in a 2:1 ratio with the aim of synthesizing [C_4_H_5_N_2_O]_2_[CuCl_4_]. However, the title compound I was obtained, which crystallizes in the triclinic system with the *P*1 space group. The copper coordination centres are similar to those described by Blanchette and Willett (1988) in [{(C_5_H_5_N)NH_2_}CuCl_3_]_2_. The H_2_O molecules and the [C_4_H_5_N_2_O]^+^ cations (having both N atoms protonated and the O atoms atom deprotonated) are packed between the anions along the *c*-axis, the water forming O—H···Cl bridges between the anions while the cations form N—H···Cl and N—H···O bonds with the anions and water molecules respectively (Fig. 2).

For related literature, see Blanchette & Willett (1988) and Furberg & Aas (1975).

### Experimental

Copper(II) chloride dihydrate and 2-hydroxypyrimidine hydrochloride in a 1:2 molar ratio were dissolved in concentrated hydrochloric acid solution. The solution was left to evaporate slowly at room temperature and resulted in the formation of green crystals after a few days.

### Refinement

H atoms bonded to O atoms were located in the difference map and refined with distance restraints of O—H = 0.84 (2) Å with *U*_iso_(H) = 1.2*U*_eq_(O). Other H atoms were positioned geometrically and refined using a riding model, with C—H = 0.93 Å and N—H = 0.86 Å, with *U*_iso_(H) = 1.2 times *U*_eq_(C, N).

### Crystal data

**Table:**

(C_4_H_5_N_2_O)_2_[Cu_2_Cl_6_(C_4_H_4_N_2_O)_2_]·2H_2_O	*Z* = 1
*M**_r_* = 762.22	*F*_000_ = 382
Triclinic, *P*1	*D*_x_ = 1.908 Mg m^−^^3^
*a* = 7.5924 (4) Å	Mo *K*α radiation λ = 0.71073 Å
*b* = 8.6401 (3) Å	Cell parameters from 10481 reflections
*c* = 10.6349 (4) Å	θ = 2.4–30.0º
α = 96.032 (3)º	µ = 2.26 mm^−^^1^
β = 100.508 (4)º	*T* = 100 (2) K
γ = 102.035 (4)º	Block, green
*V* = 663.39 (5) Å^3^	0.41 × 0.18 × 0.16 mm

### Data collection

**Table:**

Oxford Diffraction Gemini-R Ultra diffractometer	3902 independent reflections
Radiation source: fine-focus sealed tube	3269 reflections with *I* > 2σ(*I*)
Monochromator: graphite	*R*_int_ = 0.020
*T* = 100(2) K	θ_max_ = 30.1º
ω (1° width) scans	θ_min_ = 2.4º
Absorption correction: multi-scan(CrysAlis RED; Oxford Diffraction, 2007)	*h* = −10→10
*T*_min_ = 0.433, *T*_max_ = 0.71	*k* = −12→12
14528 measured reflections	*l* = −15→15

### Refinement

**Table:**

Refinement on *F*^2^	Secondary atom site location: difference Fourier map
Least-squares matrix: full	Hydrogen site location: inferred from neighbouring sites
*R*[*F*^2^ > 2σ(*F*^2^)] = 0.024	H atoms treated by a mixture of independent and constrained refinement
*wR*(*F*^2^) = 0.069	*w* = 1/[σ^2^(*F*_o_^2^) + (0.0422*P*)^2^ + 0.0753*P*] where *P* = (*F*_o_^2^ + 2*F*_c_^2^)/3
*S* = 1.12	(Δ/σ)_max_ = 0.002
3902 reflections	Δρ_max_ = 0.49 e Å^−^^3^
180 parameters	Δρ_min_ = −0.57 e Å^−^^3^
2 restraints	Extinction correction: none
Primary atom site location: structure-invariant direct methods	

### Special details

**Table:**

Experimental. CrysAlis RED, Oxford Diffraction Ltd., Version 1.171.32.5 (release 08-05-2007 CrysAlis171 .NET) Empirical absorption correction using spherical harmonics, implemented in SCALE3 ABSPACK scaling algorithm.
Geometry. All e.s.d.'s (except the e.s.d. in the dihedral angle between two l.s. planes) are estimated using the full covariance matrix. The cell e.s.d.'s are taken into account individually in the estimation of e.s.d.'s in distances, angles and torsion angles; correlations between e.s.d.'s in cell parameters are only used when they are defined by crystal symmetry. An approximate (isotropic) treatment of cell e.s.d.'s is used for estimating e.s.d.'s involving l.s. planes.
Refinement. Refinement of *F*^2^ against ALL reflections. The weighted *R*-factor *wR* and goodness of fit *S* are based on *F*^2^, conventional *R*-factors *R* are based on *F*, with *F* set to zero for negative *F*^2^. The threshold expression of *F*^2^ > σ(*F*^2^) is used only for calculating *R*-factors(gt) *etc*. and is not relevant to the choice of reflections for refinement. *R*-factors based on *F*^2^ are statistically about twice as large as those based on *F*, and *R*- factors based on ALL data will be even larger.

### Fractional atomic coordinates and isotropic or equivalent isotropic displacement parameters (Å^2^)

**Table:**

	*x*	*y*	*z*	*U*_iso_*/*U*_eq_	
Cu1	0.07237 (3)	0.45037 (2)	0.848449 (17)	0.01007 (6)	
Cl1	−0.06435 (5)	0.63425 (4)	0.75599 (3)	0.01291 (8)	
Cl2	0.19396 (5)	0.64570 (4)	1.06611 (3)	0.01065 (8)	
Cl3	0.26769 (6)	0.45986 (5)	0.70847 (4)	0.01557 (9)	
N1	0.18231 (18)	0.28152 (15)	0.92487 (12)	0.0099 (2)	
N2	0.15847 (19)	0.00585 (15)	0.91842 (13)	0.0122 (3)	
H2B	0.1020	−0.0912	0.8865	0.015*	
N3	0.69462 (19)	0.93935 (16)	0.46681 (12)	0.0124 (3)	
H3A	0.7819	1.0237	0.4760	0.015*	
N4	0.43187 (19)	0.76169 (16)	0.34665 (13)	0.0137 (3)	
H2A	0.3488	0.7298	0.2768	0.016*	
O1	0.58355 (18)	0.96484 (15)	0.25699 (12)	0.0204 (3)	
O2	−0.02211 (17)	0.10178 (14)	0.76366 (11)	0.0176 (2)	
O3	0.93114 (18)	0.22792 (15)	0.49005 (12)	0.0198 (3)	
C1	0.0983 (2)	0.12810 (17)	0.86166 (15)	0.0116 (3)	
C2	0.3215 (2)	0.30666 (18)	1.02403 (14)	0.0119 (3)	
H2C	0.3773	0.4116	1.0619	0.014*	
C3	0.3900 (2)	0.18275 (18)	1.07546 (15)	0.0132 (3)	
H3B	0.4917	0.2036	1.1436	0.016*	
C4	0.3004 (2)	0.03050 (18)	1.02078 (15)	0.0129 (3)	
H4A	0.3368	−0.0560	1.0538	0.016*	
C5	0.5717 (2)	0.89360 (19)	0.34849 (15)	0.0135 (3)	
C6	0.6850 (2)	0.85959 (19)	0.56744 (15)	0.0134 (3)	
H6A	0.7744	0.8941	0.6433	0.016*	
C7	0.5451 (2)	0.72742 (19)	0.56028 (15)	0.0141 (3)	
H7A	0.5372	0.6718	0.6301	0.017*	
C8	0.4167 (2)	0.68029 (19)	0.44567 (15)	0.0139 (3)	
H8A	0.3194	0.5917	0.4373	0.017*	
H1	0.947 (3)	0.268 (3)	0.4257 (19)	0.037 (7)*	
H2	1.024 (3)	0.267 (3)	0.551 (2)	0.044 (8)*	

### Atomic displacement parameters (Å^2^)

**Table:**

	*U*^11^	*U*^22^	*U*^33^	*U*^12^	*U*^13^	*U*^23^
Cu1	0.01130 (11)	0.00927 (9)	0.00972 (10)	0.00265 (7)	0.00141 (7)	0.00270 (7)
Cl1	0.01491 (19)	0.01236 (16)	0.01204 (17)	0.00437 (13)	0.00135 (14)	0.00458 (12)
Cl2	0.01105 (17)	0.00891 (15)	0.01027 (16)	0.00021 (13)	0.00046 (13)	0.00112 (12)
Cl3	0.01575 (19)	0.01618 (18)	0.01615 (18)	0.00296 (14)	0.00692 (14)	0.00377 (14)
N1	0.0118 (6)	0.0074 (5)	0.0097 (6)	0.0012 (5)	0.0015 (5)	0.0009 (4)
N2	0.0136 (7)	0.0069 (5)	0.0160 (6)	0.0012 (5)	0.0042 (5)	0.0008 (5)
N3	0.0102 (6)	0.0135 (6)	0.0123 (6)	0.0010 (5)	0.0007 (5)	0.0024 (5)
N4	0.0115 (6)	0.0165 (6)	0.0101 (6)	0.0018 (5)	−0.0021 (5)	−0.0012 (5)
O1	0.0218 (7)	0.0265 (6)	0.0160 (6)	0.0084 (5)	0.0052 (5)	0.0100 (5)
O2	0.0184 (6)	0.0142 (5)	0.0158 (5)	0.0017 (5)	−0.0033 (5)	−0.0013 (4)
O3	0.0198 (7)	0.0203 (6)	0.0143 (6)	−0.0049 (5)	0.0001 (5)	0.0058 (5)
C1	0.0126 (7)	0.0089 (6)	0.0133 (7)	0.0014 (5)	0.0040 (6)	0.0013 (5)
C2	0.0122 (7)	0.0106 (7)	0.0122 (7)	0.0019 (6)	0.0025 (6)	0.0010 (5)
C3	0.0140 (8)	0.0136 (7)	0.0125 (7)	0.0049 (6)	0.0019 (6)	0.0025 (5)
C4	0.0149 (8)	0.0121 (7)	0.0149 (7)	0.0058 (6)	0.0059 (6)	0.0056 (6)
C5	0.0114 (8)	0.0157 (7)	0.0141 (7)	0.0052 (6)	0.0022 (6)	0.0023 (6)
C6	0.0139 (8)	0.0137 (7)	0.0115 (7)	0.0041 (6)	−0.0005 (6)	0.0009 (5)
C7	0.0160 (8)	0.0139 (7)	0.0119 (7)	0.0028 (6)	0.0022 (6)	0.0030 (5)
C8	0.0121 (8)	0.0128 (7)	0.0158 (7)	0.0020 (6)	0.0030 (6)	−0.0004 (6)

### Geometric parameters (Å, °)

**Table:**

Cu1—N1	1.9989 (12)	N4—H2A	0.8600
Cu1—Cl3	2.2809 (4)	O1—C5	1.2119 (19)
Cu1—Cl1	2.2830 (4)	O2—C1	1.221 (2)
Cu1—Cl2^i^	2.3942 (4)	O3—H1	0.815 (16)
Cu1—Cl2	2.6093 (4)	O3—H2	0.844 (17)
Cl2—Cu1^i^	2.3942 (4)	C2—C3	1.403 (2)
N1—C2	1.314 (2)	C2—H2C	0.9300
N1—C1	1.3854 (19)	C3—C4	1.361 (2)
N2—C4	1.349 (2)	C3—H3B	0.9300
N2—C1	1.3868 (19)	C4—H4A	0.9300
N2—H2B	0.8600	C6—C7	1.373 (2)
N3—C6	1.337 (2)	C6—H6A	0.9300
N3—C5	1.387 (2)	C7—C8	1.378 (2)
N3—H3A	0.8600	C7—H7A	0.9300
N4—C8	1.335 (2)	C8—H8A	0.9300
N4—C5	1.382 (2)		
			
N1—Cu1—Cl3	88.53 (4)	O2—C1—N1	122.46 (14)
N1—Cu1—Cl1	177.38 (4)	O2—C1—N2	122.11 (14)
Cl3—Cu1—Cl1	91.956 (15)	N1—C1—N2	115.42 (13)
N1—Cu1—Cl2^i^	88.05 (4)	N1—C2—C3	123.13 (14)
Cl3—Cu1—Cl2^i^	157.928 (16)	N1—C2—H2C	118.4
Cl1—Cu1—Cl2^i^	90.538 (15)	C3—C2—H2C	118.4
N1—Cu1—Cl2	91.12 (4)	C4—C3—C2	116.78 (15)
Cl3—Cu1—Cl2	115.984 (15)	C4—C3—H3B	121.6
Cl1—Cu1—Cl2	90.982 (14)	C2—C3—H3B	121.6
Cl2^i^—Cu1—Cl2	85.883 (14)	N2—C4—C3	119.63 (14)
Cu1^i^—Cl2—Cu1	94.117 (14)	N2—C4—H4A	120.2
C2—N1—C1	120.92 (13)	C3—C4—H4A	120.2
C2—N1—Cu1	125.69 (10)	O1—C5—N4	123.48 (15)
C1—N1—Cu1	113.38 (10)	O1—C5—N3	123.19 (15)
C4—N2—C1	123.75 (13)	N4—C5—N3	113.30 (13)
C4—N2—H2B	118.1	N3—C6—C7	120.87 (15)
C1—N2—H2B	118.1	N3—C6—H6A	119.6
C6—N3—C5	123.65 (14)	C7—C6—H6A	119.6
C6—N3—H3A	118.2	C6—C7—C8	117.45 (15)
C5—N3—H3A	118.2	C6—C7—H7A	121.3
C8—N4—C5	124.63 (14)	C8—C7—H7A	121.3
C8—N4—H2A	117.7	N4—C8—C7	120.03 (15)
C5—N4—H2A	117.7	N4—C8—H8A	120.0
H1—O3—H2	109 (2)	C7—C8—H8A	120.0
			
N1—Cu1—Cl2—Cu1^i^	−87.97 (4)	C4—N2—C1—N1	−6.1 (2)
Cl3—Cu1—Cl2—Cu1^i^	−176.875 (16)	C1—N1—C2—C3	−2.4 (2)
Cl1—Cu1—Cl2—Cu1^i^	90.469 (15)	Cu1—N1—C2—C3	178.41 (11)
Cl2^i^—Cu1—Cl2—Cu1^i^	0.0	N1—C2—C3—C4	−2.5 (2)
Cl3—Cu1—N1—C2	92.08 (13)	C1—N2—C4—C3	1.5 (2)
Cl2^i^—Cu1—N1—C2	−109.72 (13)	C2—C3—C4—N2	2.9 (2)
Cl2—Cu1—N1—C2	−23.88 (13)	C8—N4—C5—O1	179.35 (16)
Cl3—Cu1—N1—C1	−87.19 (10)	C8—N4—C5—N3	−2.3 (2)
Cl2^i^—Cu1—N1—C1	71.00 (10)	C6—N3—C5—O1	−178.50 (16)
Cl2—Cu1—N1—C1	156.84 (10)	C6—N3—C5—N4	3.2 (2)
C2—N1—C1—O2	−174.50 (15)	C5—N3—C6—C7	−2.4 (2)
Cu1—N1—C1—O2	4.8 (2)	N3—C6—C7—C8	0.4 (2)
C2—N1—C1—N2	6.4 (2)	C5—N4—C8—C7	0.7 (2)
Cu1—N1—C1—N2	−174.26 (10)	C6—C7—C8—N4	0.4 (2)
C4—N2—C1—O2	174.79 (15)		

Symmetry codes: (i) −*x*, −*y*+1, −*z*+2.

### Hydrogen-bond geometry (Å, °)

**Table:**

*D*—H···*A*	*D*—H	H···*A*	*D*···*A*	*D*—H···*A*
N2—H2B···Cl1^ii^	0.86	2.56	3.4143 (14)	171
N3—H3A···O3^iii^	0.86	1.86	2.7099 (18)	168
N4—H2A···Cl2^iv^	0.86	2.30	3.1336 (14)	165
O3—H1···Cl1^v^	0.815 (16)	2.428 (17)	3.2258 (13)	166 (2)
O3—H2···Cl3^vi^	0.844 (17)	2.454 (18)	3.2653 (13)	162 (2)

Symmetry codes: (ii) *x*, *y*−1, *z*; (iii) *x*, *y*+1, *z*; (iv) *x*, *y*, *z*−1; (v) −*x*+1, −*y*+1, −*z*+1; (vi) *x*+1, *y*, *z*.
